# In vitro biological assessment of the stability of cigarette smoke aqueous aerosol extracts

**DOI:** 10.1186/s13104-020-05337-2

**Published:** 2020-10-21

**Authors:** Mark Taylor, Simone Santopietro, Andrew Baxter, Nicole East, Damien Breheny, David Thorne, Marianna Gaça

**Affiliations:** British American Tobacco, R&D, Southampton, SO15 8TL Hampshire UK

**Keywords:** In vitro, AqE, Aqueous extract, Cigarette smoke, Stability

## Abstract

**Objectives:**

Cigarette smoke aqueous aerosol extracts (AqE) have been used for assessing tobacco products, particularly with in vitro models such as oxidative stress and inflammation. These test articles can be generated easily, but there are no standardised methods for the generation and characterisation or stability. We investigated the effects of pro-oxidant smoke-derived chemicals by using 3R4F AqE generated under standardised conditioning and smoking regimes and assessed the stability over 31-week timeframe. Twenty batches generated from ten puffs per cigarette bubbled through 20 ml cell culture media were used fresh and thawed from frozen aliquots stored at – 80 ºC.

**Results:**

Nicotine levels quantified by gas chromatography/mass spectrometry and optical density at 260 nm showed chemical and physical stability from week 0 (fresh sample) to weeks 1, 4, 8 and 31 (frozen samples). No significant change in H292 human bronchial epithelial cell viability or oxidative stress were observed between fresh AqE at week 0 and frozen AqE at 31 weeks. AqEs generated by our protocol were stable for up to 31 weeks for all tested end points, suggesting that it may not be necessary to use freshly generated AqE for each study, thus reducing batch-to-batch variability.

## Introduction

Tobacco smoke is a mixture of more than 7000 constituents [1–3] partitioned in vapour and particulate phases of cigarette smoke aerosol. In vitro toxicological assessments of cigarette smoke have been conducted extensively in total particulate matter (TPM), whole aerosol, or aqueous aerosol extracts (AqE) and by a range of biological techniques [4–7]. TPM is well characterised and shows stability over 2 years [4]. Stability of whole aerosol approaches is less important for cigarettes because they are designed to generate and deliver the aerosol in seconds [5, 6]. Although the use of cigarette smoke AqE has been well documented, including the effective capturing of semi-volatiles, such as carbonyls [8–11], short-term and long-term stability of volatile and semi-volatile fractions remain uncharacterised. The generation of fresh extracts can be time consuming and subjected to a risk of batch-to-batch variability. Mitigation of variability might be possible if pooled batches are generated, stored and used throughout the lifecycle of a study.

AqE generation is further confounded by the lack of a standardised approach.

A cigarette smoke aqueous test matrix consists of one or more cigarettes smoked into a specified volume of solution and the resulting aqueous soluble fraction of the cigarette smoke (AqE) is diluted and applied to the cell surface. Studies have used multiple methods not fully described to allow replication [7, 10, 12, 13]. For instance, the puffing regime is not always specified, the capture solutions (e.g. cell culture medium) and the capture conditions (e.g. media temperature) differ, making comparisons of data difficult (Table 1).

**Table 1:** Different methods described for generation of AqE

Smoking regime	AqE generation parameters	Media conditions	Study
HCI	30 puffs/15 ml	Ice-cold DMEM	Munakata et al. [10]
Other	1 cig/10 ml	Preheated RPMI	Stabile et al. [12]
ISO	8 puffs/20 ml	Sterile PRF medium 199 (stored at – 80 °C)	Brunssen et al. [13]
NS	10 cigs/10 ml	Solvent (unspecified)	Ji et al. [14]
HCI	10 puffs/20 ml	VascuLife basal media† with supplements	Taylor et al. [7]
ISO	8 puffs/20 ml	PRF DMEM/F12	Oke et al. [15]
NS	8–10 puffs/35 ml	Saline solution	Gellner et al. [16]
NS	Smoke/25 ml	Media (unspecified)	Comer et al. [17]
NS	1 cig/10 ml	PBS	Yoon et al. [18]
NS	1 cig/10 ml	DMEM (Stored at –32 °C)	Streck et al.; Kim et al. 2002 [19]
NS	1 cig/10 ml	PBS	Nana-Sinkam et al. [20]
NS	1 cig/25 ml	RPMI-1640 (one cigarette without filter)	Kim et al. [21]
NS	2 cig/50 ml	RPMI-1640 (filters removed from two 1R4F cigarettes)	Richter et al. [22]

Conditions taken directly from source material and quoted as described. In most cases the information does not exist to replicate the exposure. †Lifeline Cell Technology, Oceanside, CA, USA

AqE, aqueous aerosol extracts; cig, cigarette; DMEM, Dulbecco's Modified Eagle Medium; F12, nutrient mixture F-12; HCI, health Canada Intense; ISO, international standards organisation; NS, not specified; PBS, phosphate-buffered saline; PRF, phenol red-free

Smoke-induced oxidative stress has been linked to chronic obstructive pulmonary disease [13, 17, 23] associated with impaired antioxidant defences and characterised by an accumulation of oxidised glutathione in the lungs of smokers. In this study, the effects of pro-oxidant smoke-derived chemicals in cigarette smoke (partitioned between the particulate and vapour phase) were investigated by use of AqE. Cigarette Smoke extracts containing both particulate and vapour phases have been shown to induce oxidative stress [24]. In addition, the stability of frozen compared with fresh AqE was assessed over 31 weeks.

## Main text

### Materials and methods

#### Study design

Cytotoxicity and oxidative stress responses were assessed by in vitro assays. Measurements of epithelial antioxidant responses to AqE exposure enabled observations of any reductions in AqE-contained oxidants, potentially due to loss of volatile or semi-volatile chemicals within the extract partitioned between the particulate and vapour phases. Viability of H292 cells following AqE exposure was quantified by cellular protease activity.

The physical and chemical stability of cigarette smoke AqE stored at – 80 °C was investigated by assessment of nicotine and tar concentrations in AqE used fresh and from frozen throughout the study. This served to assess if long-term storage of AqE resulted in significant changes in cytotoxicity and oxidative stress responses and if affected the ability to complete a full set of experiments in a study.

#### Materials and reagents

Unless otherwise stated all materials and reagents were purchased from Fisher Scientific (Loughborough, UK).

#### Test article

All cigarette smoke AqEs were obtained from Kentucky 3R4F reference cigarettes. The 3R4F is a US-blended king-sized product with a cellulose acetate filter and an International Organisation for Standardisation (ISO) tar yield of 9.4 mg per cigarette. The composition, construction and mainstream smoke chemistry yields from this product have been reported previously [25]. Prior to analysis, cigarettes were conditioned for at least 48 h at 22 ± °C in 60 ± 3% relative humidity and for no longer than 10 days, in accordance with ISO 3402:1999 [25].

#### Preparation of aqueous aerosol extracts

3R4F cigarettes were puffed on a Borgwaldt-KC RM20H rotary smoking machine (Borgwaldt, Richmond, VA, USA) using the Health Canada Intense T-115 regime (55 ml puff volume, 2 s puff duration every 30 s, and 100% ventilation blockage) [26]. Ten puffs from one 3R4F cigarette were bubbled through 20 ml cell culture media contained in an impinger per AqE. This produced a stock (100%) extract at a concentration of 0.5 puffs/ml; 20 AqE batches were produced on the same day by a single operator; all AqE batches were combined to provide a 400 ml pooled batch, which was stored at – 80 °C, except the fresh AqE aliquot used on the first day of the study. Frozen aliquots were thawed at room temperature overnight preceding the experiments and diluted with cell-type-specific culture media to the desired concentrations.

#### Dosimetry and quality control measurements

To confirm the capture of cigarette smoke constituents, assess batch-to-batch consistency of AqE, and test physical and chemical stability over time, three measurements were taken in the fresh AqE and after thawing of frozen AqE at 1, 4, 8, and 31-weeks. Nicotine concentration in the AqE was quantified via gas chromatography/mass spectrometry. An indirect measurement of nicotine concentration and the relative tar content of the extracts were also performed directly by optical density (OD) readings at 260 nm and 320 nm respectively, using a Spectramax M3 multimode spectrophotometer (Molecular Devices, San Jose, CA, USA) [7].

#### Human bronchial epithelial cell culture

Human bronchial epithelial cells (NCI-H292; American Type Culture Collection, Teddington, Middlesex, UK) were grown in cell culture flasks and maintained in RPMI 1640 medium containing sodium bicarbonate without l-glutamine, sterile-filtered and tested for endotoxins. The medium was supplemented with 10% foetal bovine serum (GE Healthcare Life Sciences, Hatfield, Hertfordshire, UK), 2 mM glutamine, 50 U/ml penicillin and 50 μg/ml streptomycin. Experimental 96-well cell culture plates were seeded with 100 µl culture medium per well at a seeding density of 1 × 10^5^ cells/ml cell suspension. Cultures were grown over 72 h at 37 °C in a humidified 5% carbon dioxide incubator prior to experimental exposures.

#### Measurement of epithelial viability

Cell viability was assessed by Apolive-Glo assay (Promega, Madison, WI, USA) in 96-well format. Viable cells were quantified through observations of live-cell protease activity. A cell permeable substrate (glycyl-phenylalanyl-amino fluorocoumarin; GF-AFC) that produces a fluorescent signal upon cleavage by viable cell proteases was co-incubated with the cells. Fluorescence signals, proportional to the viable cell number, were measured with a Spectramax M3 multimode plate reader. Exposure of cells to Triton X-100 (0.2%) allowed observations of complete cell death in the viability assay.

#### Quantification of epithelial oxidative stress

The ratio of reduced glutathione (GSH) to oxidised GSH (GSSG) was assessed in NCI-H292 cells and calculated after 4 h exposure to 100 µl AqE in a 96-well format assay (GSH/GSSG-Glo, Promega), as per the manufacturer’s protocol. Quantification of both GSH and GSSG was achieved through a GSH-dependent reaction, whereby a GSH probe, luciferin-NT (Promega), was converted to luciferin by a glutathione *S*-transferase enzyme that had been coupled to a firefly luciferase. The luminescent signal was proportional to the amount of GSH or GSSG present. Luminescent signals were measured with a Spectramax M3 multimode plate reader over a 1 s integration time. Cells were treated with potassium bromate (30 mM) to provide a pro-oxidant source required to lower the glutathione ratio, which was used as a positive control.

### Statistics

All variables were measured at weeks 0, 1, 4, 8, and 31. Statistical analysis was performed by means of ANOVA, with one-sided Dunnett’s post hoc comparisons to assess differences in dosimetry, cell viability and GSH:GSSG between experiments performed at week 0 with fresh AqE and at week 31 with frozen AqE; p values < 0.05 were considered significant. AqE were assessed at an IC_50_ to compare shifts in responses. 50% viability was represented at 56% AqE dose (Clarified in Table 2). Nicotine and OD measurements were only conducted on one occasion per assessment after the first week of the study once the variability was established. This was to ensure that the test matrix was maximised for biological analysis.

**Table 2:** Summary of findings

Analysis	Week 0 (fresh)	Week 1 (frozen)	Week 4 (frozen)	Week 8 (frozen)	Week 31 (frozen)	Conclusions
Nicotine (µg/ml)	8268 ± 635.15	7980	7250	8310	9110	No effect up to 31 weeks storage at – 80 °C
OD at 260 nm	2.26 ± 0.09	2.26	2.30	2.29	2.23	No effect up to 31 weeks storage at – 80 °C
OD at 320 nm	0.57 ± 0.02	0.57	0.58	0.57	0.58	No effect up to 31 weeks storage at – 80 °C
H292 cell viability (%; measured at 50% toxicity dose [56% AqE])	54.48 ± 4.0	56.6 ± 4.50	60.4 ± 3.3	56.9 ± 8.3	64.0 ± 2.9	No significant effect up to 31 weeks storage at – 80 °C (p = 0.138 for difference between weeks 0 and 31)
Glutathione ratio (measured at 50% toxicity dose [56% AqE])	60.25 ± 2.8	43.90 ± 5.49	43.15 ± 7.61	55.08 ± 4.25	54.47 ± 6.42	No significant effect up to 31 weeks storage at – 80 °C (p = 0.548 for difference between weeks 0 and 31)

Data are means and standard deviations (where available)

AqE, aqueous aerosol extracts; H292, Human bronchial epithelial cells (NCI-H292; American Type Culture Collection, Teddington, Middlesex, UK); OD, optical density

### Results

#### Dosimetry

**Fig. 1:**
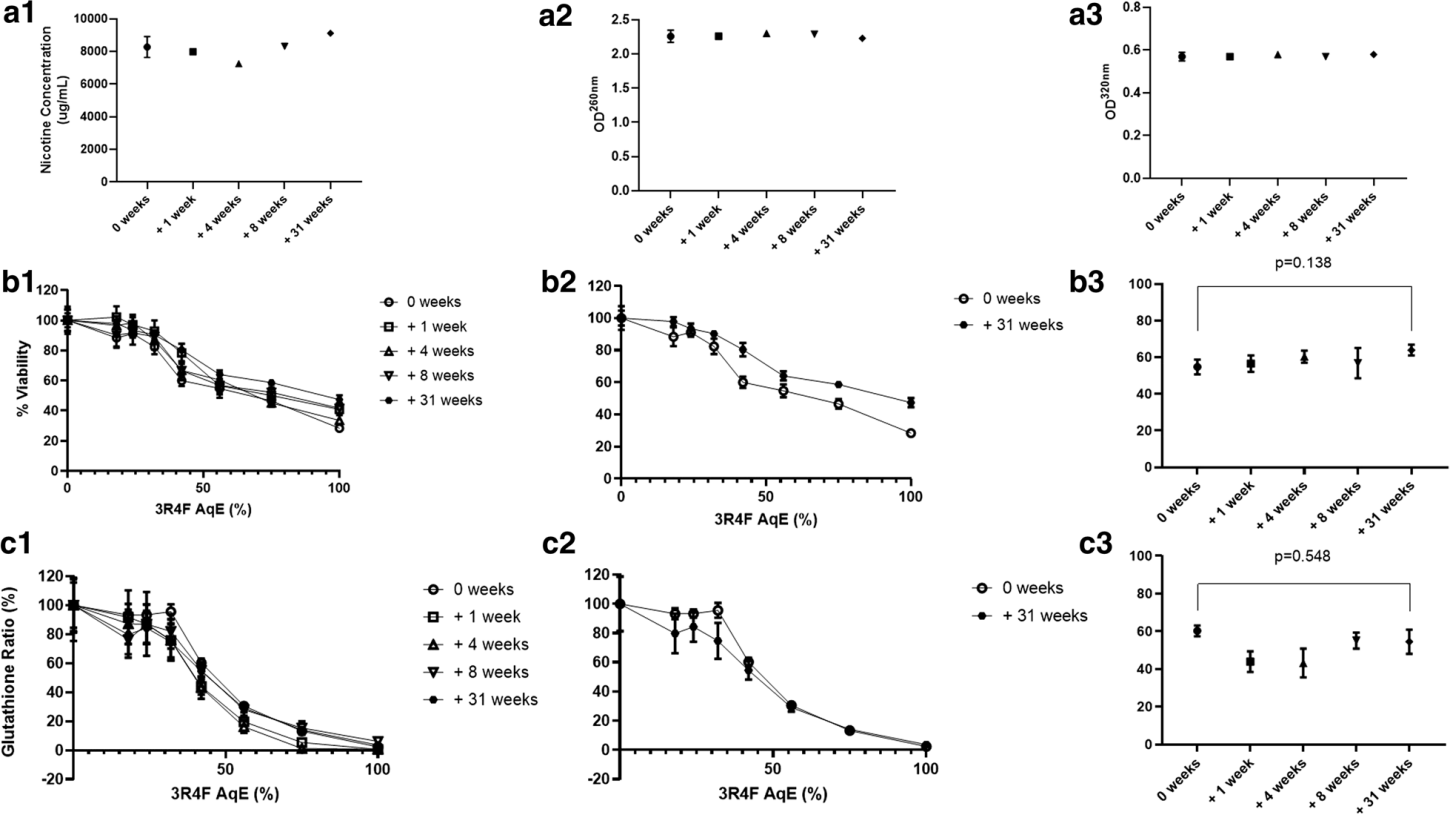
A1-3*. QC measures. (A1) nicotine, (A2) OD260nm (A3) OD 320 nm. B1-3 Cell viability (B1) All data 0–31 weeks (B2) O vs. 31 weeks comparison (B3) Data at 56% AqE dilution. C1-3* Glutathione ratio *(C1) All data 0–31 weeks (C2) O vs. 31 weeks comparison (C3) Data at 56% AqE dilution*

Storage up to 31-weeks at – 80 °C was not associated with reductions in nicotine or tar concentrations compared with fresh AqE used at week 0 (Fig. 1).

#### Cellular viability

Little variation in the results for epithelial viability was observed between fresh and frozen AqE at any dilution (Fig. 1).

#### Glutathione ratio

The ratio of GSH to GSSG at different AqE dilutions was similar for all fresh and frozen aliquots (Fig. 1).

#### Summary of findings

Storage of 3R4F AqE at – 80 °C up to 31-weeks was not associated with any deterioration in nicotine and tar concentrations or any negative effects on cell biological activity (Table 2).

### Discussion

Cigarette smoke AqE generation is complex and further confounded by differences in terminology, methodology and approaches, from study to study and between laboratories. This study addressed some of the misunderstandings and gaps in the current literature about the generation and stability of cigarette smoke AqE. The use of established methods and parameters based on published studies led to uniform generation of AqE and freezing and storing aliquots at – 80 ºC was a stable way of providing enough extract for long-term experimentation.

This methodology combined the use of an internationally recognised reference cigarette (3R4F), standard conditioning and smoking regimens [26, 27] with parameters that were the ‘average’ of those previously reported (Table 1), such as capture of ten puffs of smoke per cigarette in 20 ml cell culture media and dilution of aliquots with cell-type-specific media. This approach led to consistent generation of AqE that provided reproducibility, minimised batch-to-batch variation and allowed batches to be pooled for storage and consistent experimentation over 31-weeks.

Nicotine and tar concentrations were used as dosimetry and quality control markers for physical and chemical stability. Throughout the study, no values with frozen aliquots differed significantly from those achieved in the fresh samples. This finding indicates good stability in the storage conditions assessed. Nicotine has been reported as a stable analyte in culture media over 12 weeks at room temperature [28]. Our data supports this conclusion and suggest that nicotine concentration could be expected to remain stable for much longer than 31-weeks. OD measurements remained stable for the duration of the study.

The in vitro end points assessed determined whether biological activity would decrease after exposure to AqE stored at – 80 °C and thawed before use. The timepoints for assessment of frozen AqE (1, 4, 8 and 31 weeks) were selected to assess short-term and long-term effects of storage. Short-term effects are relevant, for example, for samples shipped to global locations, while longer-term effects account for the time that might be required to complete a full suite of experiments in a study. Both cytotoxicity and GSSG formation are thought to be driven by the semi-volatile and volatile vapour-phase fraction of cigarette smoke, which partition between the vapour and particulate phase. Analytically measuring these compounds is challenging, and reductions or shifts in analytical yields do not necessarily equate to a biological response. Therefore, cytotoxicity was investigated as a measure of AqE functionality and stability and the glutathione ratio as an indicator of oxidative stress. The results observed in fresh AqE are consistent with previously reported data [18]. After freezing, no significant differences from fresh AqE were observed up to 31-weeks.

## Conclusions

Internationally recognised conditioning and smoking regimens and the use of a reference cigarette (3R4F) allowed consistent generation of AqE. The puff number and volume captured per batch were based on data collected from the literature and averaged to align protocols. The data from multiple end points suggest that the AqE are chemically and physically stable for weeks. This study has shown up to 31-weeks, that cigarette smoke AqE is stable in respect to cytotoxicity, glutathione ratio and nicotine. Cytotoxicity and glutathione ratio can be linked to the particulate and vapour phase of cigarette smoke, so observing little to no reduction in these biological end points is reassuring for the stability of the captured chemicals with the AqE matrix. Furthermore, pooling batches and creating single-use aliquots, ensuring consistency of AqE over time in a given cell line also provides an advantage to experimental design.

### Limitations

More work is required to understand how extracts can be stored until biological activity decreases or is lost after 31-weeks. Future studies could investigate additional biological end points and more cell types, including primary cells, which might be more sensitive to subtle changes in AqE compared to immortalised cell lines. The use of more chemical markers to characterise AqE at various times would also be advantageous, especially in linking reduction in chemical stability to reduced biological responses. However, measuring these chemicals analytically at low levels and covering the required chemicals of interest could be challenging, which is why this study focused on reductions in biological effect rather than on chemical yields. Finally, this study focused on cigarette smoke extracts, however stability data are also required for tobacco heating products and electronic cigarette extracts, for which little to no information is available. This is extremely important, as the use of AqE to support testing of these novel products are increasing.

## Data Availability

Not applicable.

## Abbreviations

AqE: Aqueous extract
TPM: Total particulate matter
3R4F: Kentucky reference cigarette
GSH: Glutathione
GSSG: Oxidised glutathione
OD: Optical density (at wavelengths 260 nm and 320 nm)
HCI: Health Canada intense smoking regimen
ISO: International standard
